# Stockpiling and Comprehensive Utilization of Red Mud Research Progress

**DOI:** 10.3390/ma5071232

**Published:** 2012-07-16

**Authors:** Dong-Yan Liu, Chuan-Sheng Wu

**Affiliations:** College of Civil Engineering, Chongqing University, Chongqing 400044, China; E-Mail: cqucswu@yahoo.com.cn

**Keywords:** red mud, basic characteristics, safe stockpiling, comprehensive utilization

## Abstract

With increasing production of red mud, the environmental problems caused by it are increasingly serious, and thus the integrated treatment of red mud is imminent. This article provides an overview of the composition and the basic characteristics of red mud. The research progress of safe stockpiling and comprehensive utilization of red mud is summarized. The safe stockpiling of red mud can be divided into two aspects: the design and safe operation of the stocking yard. The comprehensive utilization of red mud can be further divided into three aspects: the effective recycling of components, resource utilization and application in the field of environmental protection. This paper points out that the main focus of previous studies on red mud stockpiling is cost reproduction and land tenure. The recovery of resources from red mud has a high value-added, but low level industrialization. The use of red mud as a building material and filler material is the most effective way to reduce the stockpiling of red mud. Red mud used for environmental remediation materials is a new hotspot and worth promoting for its simple processing and low cost.

## 1. Introduction 

Red mud is a reddish-brown colored solid waste produced during the physical and chemical processing of bauxite. Bauxite is composed of aluminum hydroxide minerals, including primarily gibbsite [Al(OH)_3_], boehmite [γ-AlO(OH)] and diaspore [α-AlO(OH)], and other compounds, such as hematite [Fe_2_O_3_], goethite [FeO(OH)], quartz [SiO_2_], rutile/anatase [TiO_2_] and kaolinite [Al_2_Si_2_O_5_(OH)_4_] [1]. The red mud, according to the production process of the aluminum, can be divided into Bayer process red mud, sintering process red mud and combined process red mud. It was reported that 0.8~1.5 t of red mud is produced by each 1 t alumina produced. Globally, the total amount of red mud produced every year is between 60 and 120 million tons [2], about 30 million tons of which is produced in China. And the accumulated quantity can reach 200 million tons in China. 

As to the treatment of red mud, the first choice of most companies from all over the world would be stockpiling it in an open yard or marine dumping. Since there is a great deal of industrial alkali, fluoride and heavy metals and other potential pollutants in red mud, long-term stockpiling of red mud would not only occupy scarce land resources, but also easily lead to serious pollution of the surrounding soil, air and groundwater. On the other hand, treatment by marine dumping may destroy the ecological balance of the ocean. The dike breach at the red mud stockpiling yard at the Ajkai Timfoldgyar Zrt alumina plant in Hungary on October 4, 2010 released between 600,000 and 700,000 m^3^ of caustic red mud suspension. This incident is unprecedented, given the scale of the release and the type of material involved [3]. And it is warning us to pay enough attention to the comprehensive treatment of the red mud. 

This article provides an overview of the basic characteristics of red mud. The main ways of comprehensive utilization are also summarized. It describes the progress of experimental research and production practice of safe stockpiling and the methods of comprehensive utilization. The aim is to provide some valuable information to further address the comprehensive utilization of red mud.

## 2. The Basic Characteristics of Red Mud

### 2.1. Chemical and Mineral Composition

There are different aluminum production processes for different bauxites that subsequently produce different types of red mud. Red mud is mainly composed of coarse sand and fine particles of mud. Its composition, property and phase vary with the origin of the bauxite and the alumina production process, and will change over time when stocked. The amount of alkali in red mud fluid is about 2 to 3 g/L (calculated by Na_2_O), which results in a pH value between 13 and 14. Table 1 and Table 2 list the chemical and mineral compositions of three kinds of red mud that are produced by the Bayer process, sintering and Bayer-sintering process. 

**Table 1:** The main chemical constituents of red mud (%) [4].

Chemical constituent	Fe_2_O_3_	Al_2_O_3_	SiO_2_	CaO	Na_2_O	TiO_2_	K_2_O	MgO	Sc_2_O_3_	Nb_2_O_5_	TREO	Loss
Bayer process	28.3	17.67	8.34	20.88	2.29	7.34	0.059	0.65	–	–	–	13.88
Combined process	10.97	7.68	22.67	40.78	2.93	3.26	0.38	1.77	–	–	–	11.77
Sintering process	6.66	9.18	18.1	38.09	4	6.72	–	–	0.02	0.0193	0.25	16.96

**Table 2:** Mineral composition of red mud (%, ω) [5].

Mineral composition (chemical formula)	Sintering process	Combined process	Bayer process
β-2CaO·SiO_2_	46	43	–
Sodium aluminosilicate (Na_2_O·Al_2_O_3_·1.7SiO_2_·nH_2_O)·NaX or Na_2_X	4	4	20
Anorthite 3CaO·Al_2_O_3_·3Si_2_O_2_ or 3CaO·Al_2_O_3_·xSiO_2_·(6-2x)H_2_O	5	2	20
Calcite CaCO_3_	14	10	19
Limonite Fe_2_O_3_·H_2_O	7	4	4
Boehmite Al_2_O_3_·H_2_O	–	1	21
Perovskite CaO·TiO_2_	4	12	15
4CaO·Al_2_O_3_·Fe_2_O_3_	6	12	–
Na_2_O·Al_2_O_3_·2SiO_2_	7	8	–
FeS_2_	1	–	–
Others	1	–	1
Total	95	96	100

It can be seen from Table 1 that the main chemical compositions of red mud are Fe_2_O_3_, Al_2_O_3_, SiO_2_, CaO, Na_2_O, TiO, K_2_O and MgO. Different kinds of red mud from the sintering process and combined process have similar composition characteristics. The contents of CaO and SiO_2_ in red mud from the sintering process and combined process are much higher than that from the Bayer process. But the contents of Fe_2_O_3_ in red mud from the sintering process and combined process are much lower than that from the Bayer process. 

It can be seen from Table 2 that the main composition of the red mud from the sintering process is β-2CaO·SiO_2_, with the mass ratio being close to 50%, the same as the combined process. And the main mineral compositions of the red mud from the Bayer process are sodium aluminosilicate, aragonite, calcite boehmite and perovskite. However, major mineral compositions in Bayer process red mud include hematite (Fe_2_O_3_), nepernepheline (including natrodavyne, katoite *etc.*), gibbsite, quartz and other phases. This is consistent with the analysis of chemical compositions as stated above. It suggests some form of calcium silicate is the primary phase. 

### 2.2. Physical Properties

Tian *et al.* [6] analyzed the mechanical property of red mud from the generation of red mud, pointing out that the properties of red mud vary significantly from different bauxites and different methods of generation. In general, red mud is a very fine material in terms of particle size distribution, having an average particle size <10 μm. Typical values would account for 90% of the volume below 75 μm. The specific surface area (BET) of red mud is as large as 64–187 m^2^·g^−1^, which indicates that red mud has a high degree of mineral particle dispersion. Red mud has a large water content, up to 700 to 1000 kg/m^3^, accounting for 79%–93% of the total weight. This water will be desorbed when the red mud gets shocked, which may lead to a decrease of mechanical properties of red mud. 

It has a porous structure with a void ratio of 2.5–3.0, a high compressibility (E_g_ = 28–40 MPa) and low shear strength (C = 9.6–74.3 kPa; φ = 13.5–21.0°). Despite red mud’s properties of high porosity and water content, it will not shrink or expand after drying [5]. The sintering process creates relatively coarse particles (0.1–0.02 mm accounting for 65%), relatively good permeability (the osmotic coefficient is 10^−4^–10^−5^), relatively easy to dehydrate and for chemical cementation, a higher cementation strength (70–100 kPa). While Bayer process red mud possesses the characteristics of fine particles (0.01–0.005 mm accounting for 65%), poor permeability (the osmotic coefficient is 10^−5^–10^−6^), difficult to dehydrate under natural conditions, difficult to use for chemical cementation, with a low shear strength (40–50 kPa), *etc.* [6,7].

## 3. Safe Stockpiling of Red Mud

### 3.1. Stockpiling Method and Design of Yard

The stocking method of red mud can be divided into two types: wet stocking and dry stocking. As to wet stocking, red mud is transported into the yard as a slurry, and then is stocked after precipitation. Contrariwise, dry stocking involves the transport of desiccative red mud into the yard, where the red mud obtains accumulation capacity by the effect of solar and air drying. Wet stockpiling capacity can be increased in this way, and it is suitable for the sintering process of red mud. But it has high requirements on the yard, especially on the initial dam, the construction and maintenance of which is costly. Because the red mud is a slurry, the dam should be more firm and impermeable. Compared with the wet stockpiling, dry stockpiling does not require such initial damming, causes little pollution, and is suitable for stockpiling Bayer process of red mud.

**Figure 1:**
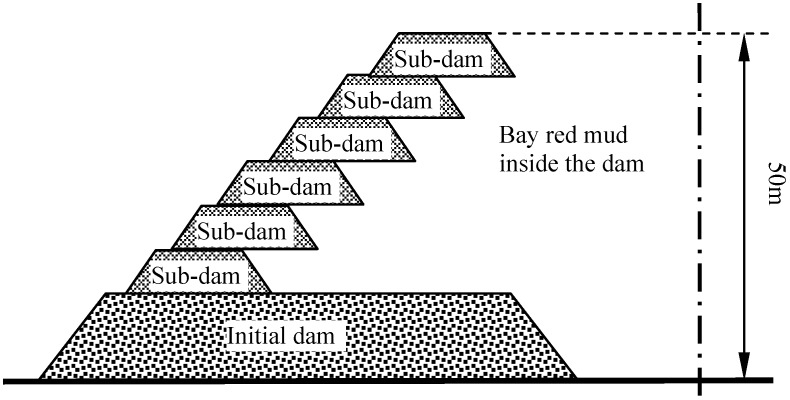
The schematic diagram of the “mixed stocking” method.

Qiao [8] and Sun [9] proposed and developed a stockpiling technique known as “mixed stocking”, which is an intermediate method between the “dry” and “wet” methods. It is a novel method using sintering red mud and Bayer red mud in the initial dam, and Bayer red mud in the sub-dams. The “mixed stocking” method combines the advantages of both, with small investment of the initial dam and sub-dam, and uncomplicated operation management. So, the design of the yard depends upon specific conditions. The schematic diagram of the “mixed stocking” method is shown as Figure 1.

### 3.2. Safety of Stockpiling Yard and Its Evaluation

As to the design of the stockpiling yard, it is necessary to recover and discharge the liquor, and improve the impermeability of the dam for the purpose of improving the safety factor of the yard and reducing the risk of pollution or dam failure. Zhou [10] suggested that increasing the quantity and quality overflow wells can improve the recovery rate of the liquor. He also indicated that well impermeability and ability to drain is conducive to keeping the dam stable. Furthermore, Wang [11] compared several drainage reinforcement methods commonly used in retention dams in particular—horizontal well drainage, radiation well drainage, light well point drainage and vertical and horizontal jointed drainage, and verified vertical and horizontal jointed drainage with a specific case study.

Li *et al.* [12] successfully applied the finite element analysis in the risk evaluation of red mud. He also discussed how finite element analysis should be used in the calculation of the saturation line and dam slope in the red mud disposal field, and proved the validity of the finite element numerical method with the safety assessment for a real yard capacity improvement case study. Rao [13] discussed the causes of cracks appearing in dry red mud, pointing out that factors such as settlement differences, dehydration shrinkage, dissolution of soluble salts and pressure differences led to the formation of cracks. Cracks bring potential danger to the integrity, impermeability and safe operation of yard. Research about crack formation mechanism, the crack growth mechanism and the regulation to prevent crack formation have very important significance, and thus require more attention.

## 4. Comprehensive Utilization of Red Mud

The key to solving red mud stockpiling is to develop a comprehensive utilization technology that consumes red mud or converts it into a secondary resource. Since the 1950s, scientists have carried out research projects that explore disposal and utilization of red mud, according to the unique physical and chemical properties of red mud, which can be divided into three categories. First, recovery of the useful component metals in red mud. Second, reuse of red mud as raw materials, especially for cement production. Third, application of red mud for environmental protection, such as adsorption to purify water.

### 4.1. Recovery of Components in Red Mud 

Red mud primarily contains elemental compositions such as Fe_2_O_3_, Al_2_O_3_, SiO_2_, CaO, Na_2_O and K_2_O. Besides, it also contains other compositions, such as Li_2_O, V_2_O_5_, TiO_2_ and ZrO_2_. For instance, the content of TiO in red mud produced in India can be as much as 24%. Because of the huge amount of red mud, value elements like Ga, Sc, Nb, Li, V, Rb, Ti and Zr are valuable and abundant secondary resources. Therefore, it is of great significance to recover metals, especially rare earth elements, from red mud.

#### 4.1.1. Recovery of Divalent Metals in Red Mud

Due to the characteristics of a high iron content, extensive research into the recovery of iron from Bayer process red mud have been carried out by scientists all over the world. The recycling process of iron from red mud can be divided into roasting magnetic recovery, the reducing smelting method, the direct magnetic separation method and the leaching-extraction method, according to the different ways of iron separation. Researchers in Russia, Hungary, America and Japan have carried out iron production experiments from red mud. Researchers from the University of Central South have made steel directly with iron recovered from red mud [14]. The Chinese Metallurgical Research Institute has enhanced the iron recovery rate to 86% through making a sponge by red mud—magnetic separation technology. Sun *et al.* [15] researched magnetic separation of iron from Bayer red mud and determined the process parameters of the magnetic roasting-magnetic selecting method to recover concentrated iron ore.

In consideration of high content of aluminum and sodium in the red mud, only by recovering them can we make full use of these resources. Zhong *et al.* [16] recovered A1_2_O_3_ and Na_2_O in red mud by the Sub-Molten Salt Method, with a one-way A1_2_O_3_ recovery rate of 88%. After dealuminization, the red mud undergoes a deep sodium removal treatment by NaOH solution, recycling Na_2_O from red mud. Zheng *et al.* [17] discussed an aluminum and sodium recovery process of a soda lime method after adding silicon slag into red mud. Under the optimum conditions, the dissolution rates of aluminum and sodium is up to 95%, with red mud after the dissolution a Na_2_O content of less than 1%, meeting the requirements for cement materials.

#### 4.1.2. Recovery of Rare Earth Elements in Red Mud

Ochsenkühn-Petropulu *et al.* [18] compared metal leaching recovery under different concentrations of solvent and different leaching conditions. Their results showed that the leaching recovers scandium, yttrium, heavy rare earth elements, middle rare earth elements and light rare earth elements at the rates of 80%, 90%, 70%, 50% and 30% respectively. They also indicated that, under the leaching conditions, a concentration of 0.5 mol/L, a temperature of 25 °C, leaching time is 24 h and a solid-liquid ratio of 1:50, the leaching rate is as follow: nitric acid > hydrochloric acid > sulfuric acid. In another study of Ochsenkühn-Petropulu *et al.* [19], the ion exchange-solvent extraction method was used to extract scandium, yttrium and lanthanides. The process leaches the ions after mixing red mud and nitric acid (0.6 mol/L) in a liquid-solid ratio of 200:1 and stirring them for 1 hour at room temperature and at atmospheric pressure. The final leaching rates can be 50%–75%. Smirnov *et al.* [20] developed a new process for the recovery and gathering of scandium, uranium and thorium from red mud slurry by the resin adsorption—dissolving process, recovering scandium at a rate of 50%.

Xue *et al.* [21] recovered scandium from Bayer process red mud by the roasting-acid leaching method, with a scandium leaching rate of more than 80%. They calcined red mud to remove water and then leached Sc with sulfuric acid solution, leaving the impurities remaining in the residues. Zhang [22] recovered the metal ions Ti, Sc, Fe and Al by the double acid leaching method from Bayer process red mud. The first acid leaching of red mud is to recover Sc, Fe and Al from red mud by adding low concentrations of hydrochloric acid. The second acid leaching is to decompose the Ti-rich residues of the first leaching, add water, and recover Ti from the decomposed solution, with a rate of more than 98%. Wang [23] studied the extraction of Sc from red mud, and obtained a final product purity of 95% by using hydrochloric acid as leaching agent, with a liquid-solid ratio of 5:1, a reaction temperature of 60 °C and a reaction time of 1 h. Chen [24] studied the separation of vanadium by the method of precipitation from Bayer process sodium aluminates solution, with a total V_2_O_5_ recovery rate of 45%.

### 4.2. Resource Utilization of Red Mud

As to the resource utilization of red mud, alumina companies have been carrying out many technical researches on production of construction material, especially cement production and glass production, production of filling material for plastic, production of road base. And they have made some progress, especially in the production of cement using red mud.

#### 4.2.1. Production of Construction Materials from Red Mud

##### 4.2.1.1. Cement

Dicalcium silicate in red mud is also one of the main phases in cement clinker, and red mud can play the role of crystallization in the production of cement clinker. Fly ash is mainly composed of SiO_2_ and Al_2_O_3_, thus can be used to absorb the water contained in the red mud and improve the reactive silica content of the cement. Scientists conducted a series of studies into the production of cement using red mud, fly ash, lime and gypsum as raw materials. Use of red mud cement not only reduces the energy consumption of cement production, but also improves the early strength of cement and resistance to sulfate attack [25].

Ekrem Kalkan [26] studied using red mud as a cement stabilizer. In 1980, Barsherike [27] studied the possibility and rationality of producing cement with red mud as the raw material component of Portland cement, and successfully prepared cement complying with the relevant standards. Vangelatos [28] studied the preparation of ordinary Portland cement from red mud, lime and freestone, and the 28-day compressive strength of the cement strength can reach 63MPa.

In China, research has been completed on the production of sulfo—aluminate cement from red mud in 1955 [29]. This kind of production process is simple and inexpensive. However, the performance of the cement, with the exceptions, which may be considerable, of some individual indicators such as soundness, is close to or greater than ordinary Portland cement. Pan *et al.* [30] studied slag and red mud activated by a composite solid alkaline activator, and developed alkali slag red mud cement which has the properties of greater early strength (the initial and final setting is separately 62 min and 95 min), high compressive strength (the 28-day compressive strength can be up to 125 MPa) and excellent resistance to corrosion, utilizing 30% of the red mud. Liang [31] and Zhong [32] prepared cement—red mud concrete using red mud. The compressive and flexural strength of this kind of concrete is close to or even higher than that of ordinary concrete, meeting the requirement of cement concrete used for pavement materials (the 28-day compressive strength is about 30–40 MPa; the 28-day flexural strength is about 4.5–5.5 MPa).

##### 4.2.1.2. Brick

As an alternative to traditional raw materials used in brick production, red mud utilization can not only reduce the cost of raw materials, but also have great environmental significance. Xing [33], Yang [34], Zhang [35], Nevin [36] *et al.* separately reported the production of non-steam-cured and non-fired brick, fly ash brick, black pellet decorative brick and ceramic glazed tile. For instance, non-steam-cured and non-fired brick is developed by using industrial residues as raw materials, by adding cement and lime as binder and by pressing and natural curing technology. The Institute of Shandong Aluminum Company and the Institute of Chinese Great Wall Aluminum Company separately achieved the production process of non-steam-cured and non-fired brick using red mud and fly ash as raw materials. The active constituents, SiO_2_ and CaO, respectively accounting for 70% in sintering process red mud and 80% in fly ash, are, from the aspects of cost and performance, the ideal raw materials for the production of non-steam-cured and non-fired brick.

##### 4.2.1.3. Glass

Yang *et al.* [37] conducted an experiment for red mud-fly ash glass, in which the maximum content of red mud and fly ash is collectively more than 90%. They acquired the optimum heat treatment process through investigation of crystallization and the factors influencing the crystal nucleation and growth. With red mud and chromium slag as the main materials, and quartz sand, fluorite, toner, manganese slag and other substances as the auxiliary materials, Liang *et al.* [38] successfully produced black glass decorative materials, which have good mechanical strength, chemical stability and optical properties.

##### 4.2.1.4. Aerated Concrete Block

Aerated concrete is a new light porous building material that has great performances such as thermal insulation, fire resistance and seismic resistance, and is made from calcareous and siliceous materials. Red mud aerated concrete, developed by using cement (15%), lime (12%–15%), red mud (35%–40%) and silica sand (33%–35%), has the compressive strength and bulk density, complying with the lowest intensity level (MU7.5) of Chinese standards—about the strength of concrete block [39]. But, its production process is basically the same as that used to produce other aerated concrete. So, this process can reduce the costs of the production of aerated concrete by taking advantage of red mud. It is said that this process will become one of the new methods of red mud utilization.

#### 4.2.2. Utilization of Red Mud As Filling Material

##### 4.2.2.1. Road base Material

High-grade road base material using red mud from the sintering process is promising, that may lead to large-scale consumption of red mud. Qi [40] suggest using red mud as road material. Based on the work of Qi, a 15 m wide and 4 km long highway, using red mud as a base material, was constructed in Zibo, Shandong Province. A relevant department had tested the subgrade stability and the strength of road, and concluded that the red mud base road meets the level I standards of lime industrial waste stabilized soil and meets the strength requirements of the highway [41].

##### 4.2.2.2. Mining

Yang *et al.* [42], from the Institute of Changsha Mining Research, have studied the properties, preparation and pump pressure transmission process of red mud paste binder backfill material. Based on this study, a new technology named “pumped red mud paste cemented filling mining” has been developed by the Institute of Changsha Mining Research, in cooperation with the Shandong Aluminum Company. They mixed red mud, fly ash, lime and water in a ratio of 2:1:0.5:2.43, and then pumped the mixture into the mine to prevent ground subsidence during bauxite mining. The tested 28-day strength can reach to 3.24 MPa. This technology is a new way not only for the use of red mud, but also for non-cement cemented filling, successfully resolving the problem of mining methods in the Hutian bauxite stope. Underground exploitation practice on the bauxite has proved that cemented filling technology is reliable and can effectively reduce the filling costs, increase the safety factor of the stope and increase the comprehensive benefits of mining [43].

##### 4.2.2.3. Plastic

For PVC (polyvinyl chloride), red mud is not only a filler that has a reinforcing effect, but is also an efficient and cheap thermal stabilizer, providing the filled PVC products with an excellent anti-aging property. Its lifetime is 2 to 3 times that of ordinary PVC products. At the same time, the fluidity of red mud is better than other fillers, which makes it plastic with good processing properties. And the red mud PVC composite plastics have fire retardant property, and can be made into red mud plastic solar water heaters and plastic construction profiles [44].

### 4.3. Application of Red Mud in the Field of Environmental Protection

#### 4.3.1. Wastewater Treatment by Red Mud

With the development of industry, the amount of industrial wastewater discharged rises sharply, causing more pollution of water bodies such as rivers and lakes and the pollution of the surrounding ecological environment. The surface reactivity of the oxide minerals in red mud promote the mobilization and adsorption of heavy metal ions like Cu^2+^, Pb^2+^, Zn^2+^, Ni^2+^, Cr^6+^ and Cd^2+^ from the water body, thereby reducing the degree of water pollution. Therefore, dealing with wastewater with red mud is a promising technology, and there are a lot of reports around the world [45]. 

##### 4.3.1.1. Heavy Metal Ions

Lopez [46] made an aggregate of red mud and 8% anhydrite and studied its adsorption property for Cu^2+^, Zn^2+^, Ni^2+^, Cd^2+^ and other heavy metal ions. The 48 h maximum adsorptions were respectively 19.72 mg/g, 12.59 mg/g, 10.95 mg/g and 10.57 mg/g. Vaclavikova [47] studied the influence of pH on the absorption efficacy of red mud for Ca^2+^, Zn^2+^ and other ions in wastewater. The result showed that the adsorption capacity when the pH is 7 is twice as that when the pH is 6. Erdem [48] and Santona [49] studied the adsorption of activated red mud for heavy metal ions, and the results showed that the adsorption rate of activated red mud for heavy metals was significantly increased.

However, due to the fact that red mud contains a variety of metal ions, it has a risk of introducing new contamination during the applications of red mud on absorption of metal ions. It should be noted that this technique is not perfect and demands more in-depth studies.

##### 4.3.1.2. Non-metallic Ions

Cengeloglu [50] studied the adsorption capacity of red mud in the solution state for fluoride and NO_3_^−^ ion in water after the acid activated treatment, which is meant to reduce the pH of the red mud by adding acid and improve the reacting activity of red mud. Akay [51] and Li [52] *et al.* successfully used red mud as adsorbent to remove phosphate from water, with an adsorption rate more than 90% when the pH is 6.2. Ahundogan’s study [53] showed that acid-activated red mud led to removal efficiencies of As (V) and As (III) in water up to 97% and 88%. Namasivayarn [54] made use of red mud to remove Congo red in textile dye wastewater. He investigated the influence of pH and adsorbent content on the adsorption efficiency and indicated that, when the pH is 2.0, the absorption rate can reach a maximum value of 82%. Wang *et al.* [55] studied the influence of thermal activation and acid treatment on absorption onto red mud for dye and methylene blue from wastewater, and confirmed that these treatments can enhance the absorption rate about 5 times.

#### 4.3.2. Soil Improvement of Red Mud

Red mud has a favorable environmental repair effect on the soil that has been contaminated by heavy metal elements [56]. One of the explanations for the mechanism is that red mud can absorb heavy metal ions such as Cu^2+^, Ni^2+^, Zn^2+^, Pb^2+^, Cd^2+^, Cr^6+^, Mn^4+^, Co^3+^ and Hg^2+^ in the soil; the form of heavy metal ions changes from exchangeable ions into bonding oxides. Another mechanism is the precipitation reaction of carbonate in red mud with the heavy metal ions, and that causes these ions to deposit. In turn, the activity and reactivity of heavy metal ions in the soil are reduced, microbial activity and plant growth are promoted. Gao *et al.* [57] conducted some studies and showed that red mud can significantly decrease Cd and Zn at the exchangeable state or effective state in the soil.

Ciccu R. [56] used red mud to modify soil polluted by heavy metal. The result showed that red mud can reduce the heavy metal content in seriously polluted soil and reduce the absorbed dose of heavy metal. Lombi [58] found that adding 2% of red mud to the soil restrained the absorption of crops for Cu^2+^, Ni^2+^, Zn^2+^, and Cd^2+^. After the modification, the mass concentrations of Zn in the soil pore water and lettuce body decreased respectively by 95% and 97%.

#### 4.3.3. Treatment of Waste Gas Containing Sulfur by Red Mud

Bekir *et al.* [59] activated red mud by drying and roasting, and studied the absorption of this activated red mud for SO_2_ which accounts for about 18% of the volume of the gases emitted from manufacturing chimneys. The desulfurization rate is initially 100%, and is still as high as 94% after 10 cycles. Chen *et al.* [60] carried out research on the absorption and purification of waste gas containing SO_2_ by red mud. They pointed out that the adsorption by Bayer red mud is a process of chemical reaction and physical adsorption, and that it requires small particle sizes (more than 50% of the particles are smaller than 45 μm) and a large specific surface area (10~20 m^2^/g) to improve the speed and depth of the chemical reaction, determining that the Bayer process red mud is a excellent absorbent for SO_2_. The main chemical reactions are as following:

SO_2_(g)+ Na_2_O→Na_2_SO_3_ (1)

4SO_2_(g)+4Na_2_O→3Na_2_SO_4_+Na_2_S (2)

4.5SO_2_(g)+Al_2_O_3_→Al_2_(SO_4_)_3_+1.5S (3)

4SO_2_(g)+4CaO→3CaSO_4_+CaS (4)

Reserves of high grade and high-sulfur bauxite are rich in China. The roasting pretreatment process for desulfurization will generate a lot of SO_2_ gas. Lv *et al.* [61], when studying high-sulfur bauxite, put forward a new roasting pretreatment technology that absorbs the acidic SO_2_ exhaust generated by the roasting process directly by alkaline red mud generated in the alumina production process. This technology can simultaneously solve the problems of the absorption of SO_2_ exhaust and the neutralization reaction of alkali red mud. What is more, the modified neutral or alkalescent red mud can also be used as construction material.

## 5. Conclusions

Overall, the comprehensive utilization of red mud generated in the process of industrial production of alumina is still a worldwide problem. At current levels technology and practice, the capacity of consumption and secondary utilization is seriously insufficient. 

The secure stockpiling of red mud has to see a reduction of stockpiling costs and improvement of efficiency. So stockpiling is not a fundamental way to resolve the problems of red mud. Only through economical and viable comprehensive utilization can people resolve them effectively in the long term.

As to the recovery of components from red mud, there are a lot of problems making for significant increases in recycling process costs and energy consumption, becoming serious impediments to industrial development. Therefore, we need to promote the industrialization of precious metal recovery processes, optimize complex processes and develop new ones.

Although the added value is relatively low, the resources utilization of red mud is the most widely used way and the most effective way to resolve the red mud stockpiling problem. Red mud can also be used to produce other construction materials. A mature, relevant technology would greatly promote the consumption of red mud.

Applying red mud as an environmental remediation material is a new hot point in terms of utilization. Due to the simple process, low cost, it is worth promoting its application in the field of environmental protection. However, there is a risk of introducing new contamination, and a difficulty of recycling it after the application. Therefore, more in-depth studies are needed and a comprehensive assessment of chemical and biological effects.
